# Superhydrophobic Drag-Reduction Spherical Bearing Fabricated by Laser Ablation and PEI Regulated ZnO Nanowire Growth

**DOI:** 10.1038/s41598-017-05546-z

**Published:** 2017-07-20

**Authors:** Rui Weng, Haifeng Zhang, Yanjing Tuo, Yang Wang, Xiaowei Liu

**Affiliations:** 1 MEMS Center, Harbin Institute of Technology, Harbin, 150001 PR China; 2 State Key Laboratory of Urban Water Resource & Environment (Harbin Institute of Technology), Harbin, 150001 China

## Abstract

The resistance of the bearing is a significant factor affecting the performance of the ball-disk rotor gyroscope. The micro and nano combined surface with low surface energy material modifications can be hydrophobic. This can reduce the drag when the bearing is lubricated by deionized water. Laser ablation method is utilized to form micron-scaled structures on the surface of the stainless steel rotor ball. And the nanostructures are formed by PEI (Polyetherimide) regulated ZnO nanowires growth. After low surface energy material modification, the water contact angle of processed surface was 163° and the sliding angle was less than 4°. The maximum rotational speed was enhanced by up to 82.77% at 1.5 W driving power. Experiments show that the superhydrophobic drag-reduction spherical bearing has good short-term reliability. At 5 V drive voltage, the bearing can extend the rotational speed of ball-disk rotor gyroscope to 35000 rpm, and maintain the normal operation for longer than 40 minutes. This is quite meaningful for short-term-work or one-time-use rotor gyroscopes.

## Introduction

**Figure 1:**
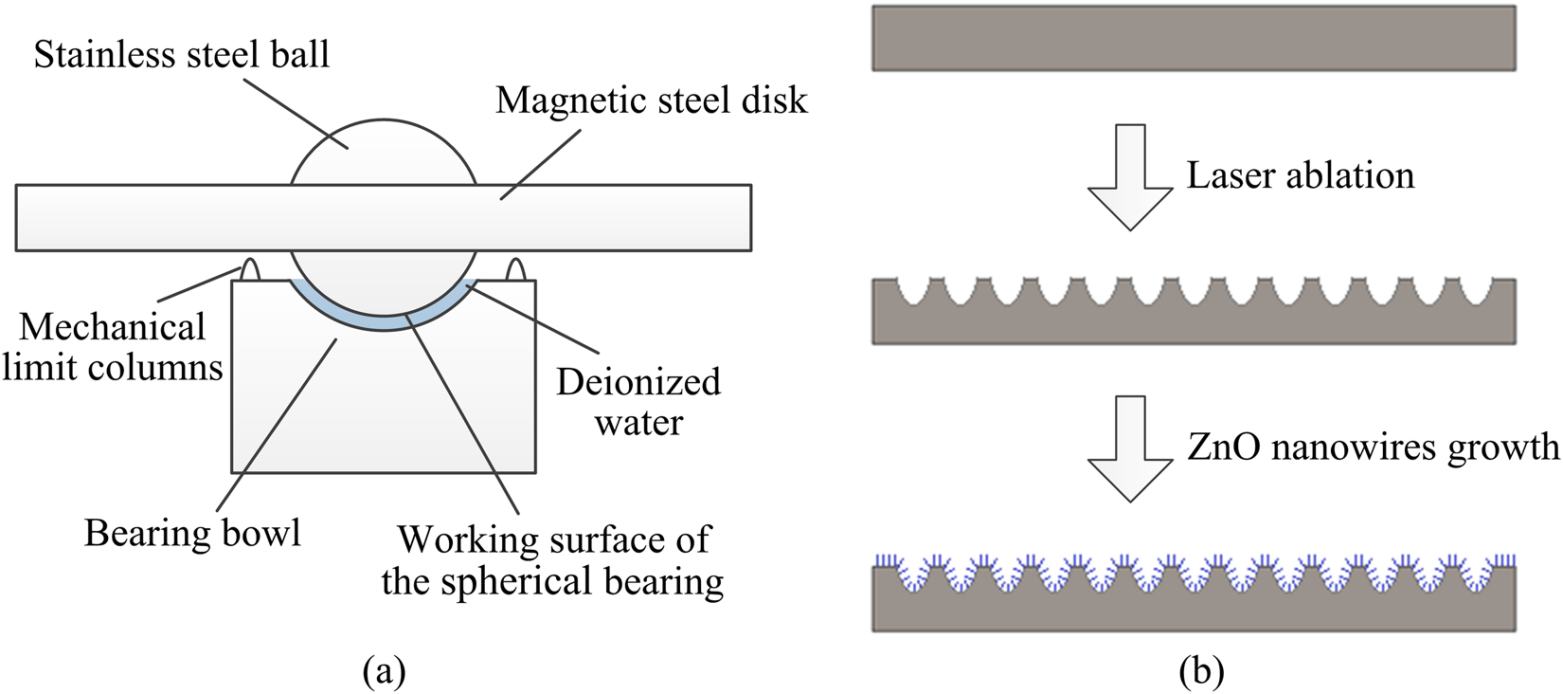
(**a**) The structure of the spherical bearing showing the working surface. (**b**) The fabricating method of the micron-nano composite structures.

Rotor gyroscopes are sensors of small volume and high accuracy. They are widely used in inertial navigation systems^1, 2^. For the rotor gyroscope works on the Coriolis force, the rotational speed of the rotor is one of the decisive factors directly affecting the sensitivity^3–5^. To improve the performance without increasing the driving power consumption, the resistance torque of the bearing should be reduced. Generally, rotor gyroscopes mostly use rolling ball bearings. When the size of a rotor gyroscope is reduced, the rolling ball bearing is no longer the best choice due to accuracy and vibration^6, 7^. Accordingly, a novel spherical bearing based on the rotor body was proposed, as is shown in Fig. 1a. For the direct solid-to-solid sliding surface increases the starting resistance and also leads to the bearing wear^8^, the mechanical limit columns are added to the bearing bowl to prevent direct solid-to-solid contact on the working surface of the spherical bearing. Now, the key problem of this spherical bearing is the viscous force of the water slick produces much resistance at a high rotational speed, and limits the further improvement of the performance of the device^9, 10^.

Studies have shown that superhydrophobic surfaces have drag-reduction effects^11–21^. In this work, we proposed a novel method to lower the resistance torque of spherical bearings in rotor gyroscopes based on this theory. Firstly, we use precision ultraviolet laser processing technology to form micron scaled structures^22–26^. Then use two-step ZnO nanowires growth method to develop micron-nano composite structures^27–34^, as is shown in Fig. 1b. The samples with ZnO nanowires were then immersed in the fluorinating agent for low surface energy modification^21, 35–38^.

After this process, the sample surfaces showed superhydrophobic characteristics. The water contact angle is 163°, while the sliding angle was less than 4°. In order to verify the drag reduction effect and the reliability of the bearing, we apply this treatment to the working surface of the bearing ball. The experimental results show that the processed surface can significantly reduce the friction of the spherical bearing. With the help of this technique, the maximum rotational speed of the rotor can be increased by up to 82.77%, and thus greatly improve the sensitivity and stability of the device. When the rotational speed is 45000 rpm and the driving power limit is 1.5 W, the spherical bearing with drag-reduction structures can work continuously for about two hours. It showed good short-term reliability in the durability test. This processing method can be used in short-term or one-time use devices to enhance their performance.

## Results and Discussion

### Surface morphology

**Figure 2:**
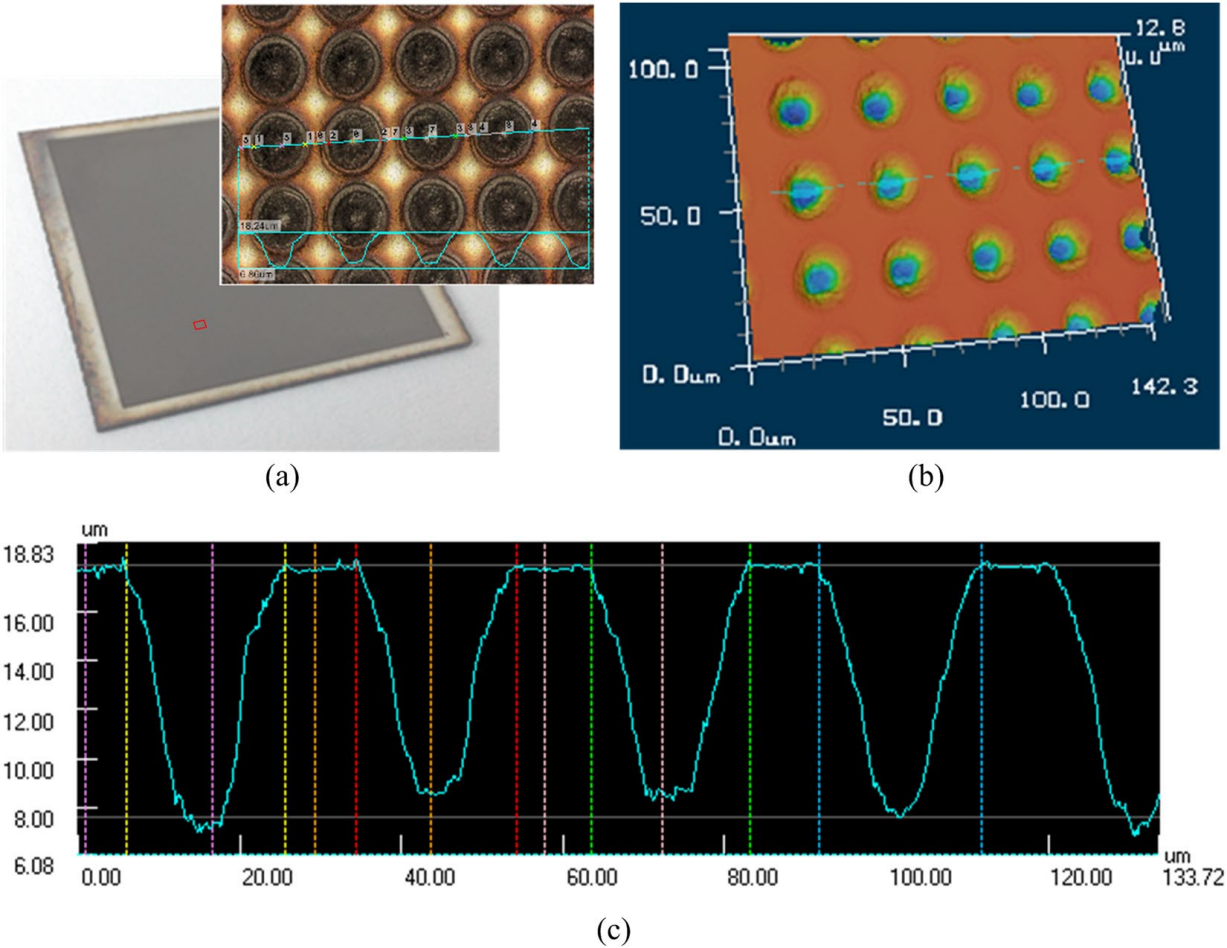
The morphology of the laser ablated surface: (**a**) The optical image and 2D topography map of the scanned area. (**b**) The 3D image of a laser ablated stainless steel foil. (**c**) The cross-section profiles along the radial direction of the microwells.

**Figure 3:**
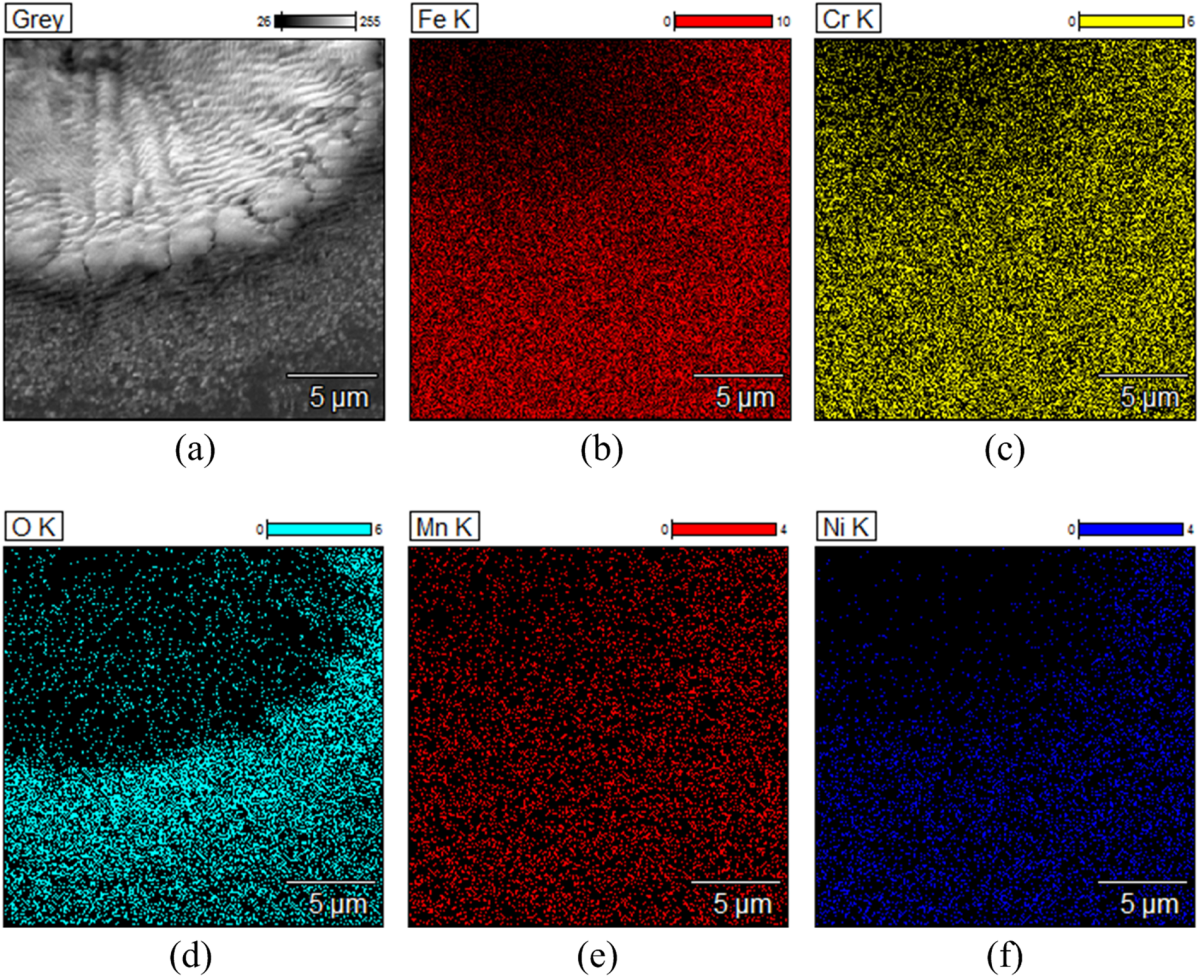
The SEM image and EDS image showing the distribution of elements.

By visual observation, the laser ablated portion of the stainless steel surface became darker. To learn more about the surface topography after laser ablation, the surface was further studied by 3D digital video microscope. The optical image and 2D topography map of the scanned area are shown in Fig. 2a. Figure 2b,c show the corresponding 3D image and the cross-section profiles along the radial direction of the microwells. It can be seen from the figures that the micron structure induced by laser ablation method were arranged in neat rows with a uniform depth of around 10μm, and the diameter of microwells obtained was 20 μm. Through the SEM image (Fig. 3a), it can be seen that there were closely spaced granular structures on a-hundred-micron scale in the ablated parts. Since the laser etching process is carried out in air, the edge of the slag is partially oxidized under high temperature. This can also be confirmed by the energy-dispersive X-ray spectroscopy (EDS) images shown in Fig. 3b–f. The EDS images show that there is a significant amount of oxygen element present there, as is shown in Fig. 3d.

**Figure 4:**
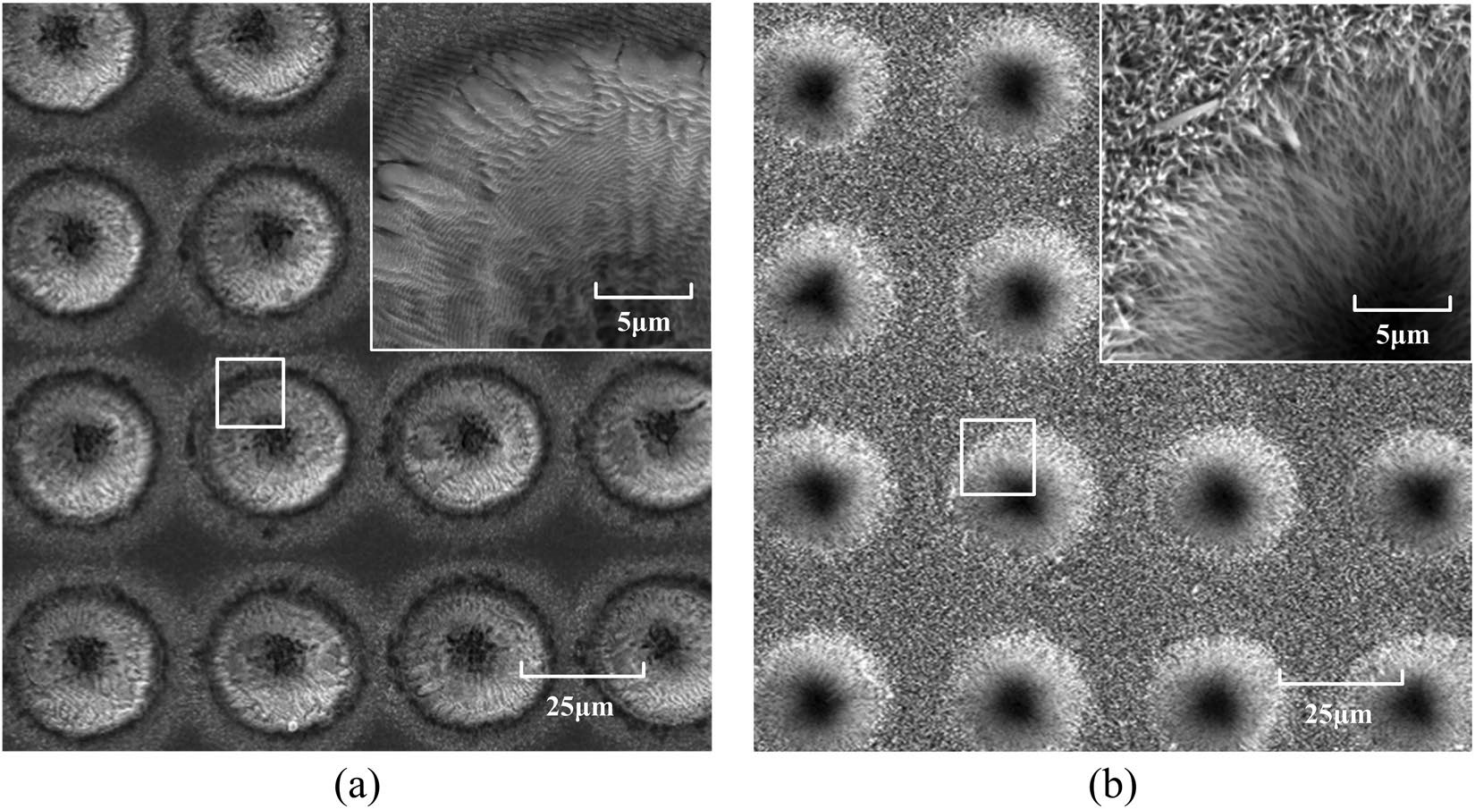
The SEM images of the sample before (**a**) and after (**b**) ZnO nanowires growth.

**Figure 5:**
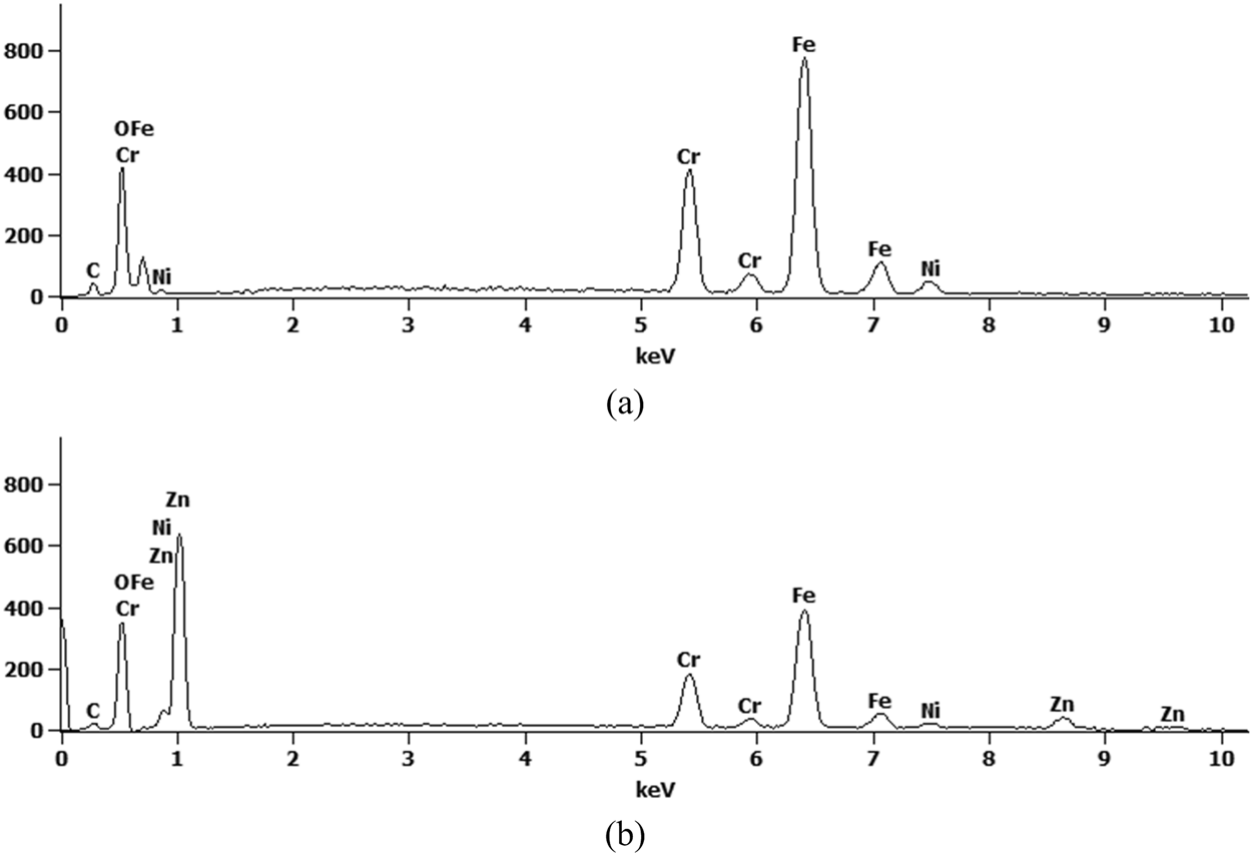
The EDS spectrums of samples before (**a**) and after (**b**) ZnO nanowires growth.

Vertically aligned ZnO nanowire arrays were obtained via liquid-phase deposition (LPD) method. Through the SEM images in Fig. 4a,b, it can be seen that the sample surface including the inner parts of the microwells was uniformly covered with a layer of nanograss. The changes in chemical composition of the sample surface before and after the process can be observed through the EDS spectrums, as is shown in Fig. 5a,b. Through EDS spectrums, we can confirm that the composition of the nanograss is zinc oxide.

### Mechanism of ZnO nanowires growth

The growth process of ZnO nanowires can be divided into two steps. The first step is the preparation of the seed layer, while the second step is the growth of ZnO nanowires based on the seed layer.

When the stainless steel foil stained with zinc acetate solution is heated at 120 °C, the solution was evaporated to dryness, with the formation of hydrated zinc acetate. Then hydrated zinc acetate lost crystal water, generating zinc acetate. When the samples were heated on the electric heating plate at 370 °C, zinc acetate decomposes to produce zinc oxide, acetone and carbon dioxide. At the reaction temperature, the latter two are both gases. They evaporated into the air, leaving ZnO with directional properties. The ZnO seed layer was generated.

This process can be described by the following two chemical equations:

$${\mathrm{Zn}(\mathrm{CH}}_{{\rm{3}}}{\mathrm{COO})}_{{\rm{2}}}\cdot {{\rm{2H}}}_{{\rm{2}}}{\rm{O}}\mathop{\longrightarrow }\limits^{{\rm{120}}^\circ {\rm{C}}}{\mathrm{Zn}(\mathrm{CH}}_{{\rm{3}}}{\mathrm{COO})}_{{\rm{2}}}+{{\rm{2H}}}_{{\rm{2}}}{\rm{O}}\uparrow $$

$${\mathrm{Zn}(\mathrm{CH}}_{{\rm{3}}}{\mathrm{COO})}_{{\rm{2}}}\mathop{\longrightarrow }\limits^{370^\circ {\rm{C}}}{\rm{ZnO}}+{{\rm{CH}}}_{{\rm{3}}}{{\rm{COCH}}}_{{\rm{3}}}\uparrow +{{\rm{CO}}}_{{\rm{2}}}\uparrow $$

Then the samples were put into the preheated reaction solution consisting of zinc nitrate, Hexamethylenetetramine and PEI solution for self-assembled ZnO nanowires growth. This is a typical LPD process; the chemical reactions in the solution can be described by the following equations:

$${{(\mathrm{CH}}_{{\rm{2}}})}_{{\rm{6}}}{{\rm{N}}}_{{\rm{4}}}+{{\rm{6H}}}_{{\rm{2}}}{\rm{O}}\to {\rm{6HCHO}}+{{\rm{4NH}}}_{{\rm{3}}}$$

$${{\rm{NH}}}_{{\rm{3}}}+{{\rm{H}}}_{{\rm{2}}}{\rm{O}}\to {{{\rm{NH}}}_{{\rm{4}}}}^{+}+{{\rm{OH}}}^{-}$$

$${{\rm{2OH}}}^{-}+{{\rm{Zn}}}^{{\rm{2}}+}\to {\mathrm{Zn}(\mathrm{OH})}_{{\rm{2}}}\to \mathrm{ZnO}(s)+{{\rm{H}}}_{{\rm{2}}}{\rm{O}}$$

In the reaction process, PEI in the solution can be complexed with Zn^2+^, thereby regulating the precipitation rate of Zn^2+^. Hexamethylenetetramine acts not only as a homogeneous precipitant, but also an organic coating agent. During the zinc oxide precipitation process, it can control the reaction rate while adjust the grain growth direction simultaneously, forming the grass-like nanowires.

### Wettability analysis

According to the literatures, there are two possible forms of hydrophobic surface, namely Wenzel state and Cassie state^12–14, 21^. Wenzel state is a high adhesion state. The contact angle is increased by increasing the solid-liquid contact area. While Cassie state is a low adhesion state. The contact angle is increased by reducing the solid-liquid contact area. In macroscopic conditions, the droplets are easy to roll away from the contact surface in Cassie state. This non-wetting characteristic is expected to reduce the drag of the solid-liquid contact surface.

**Figure 6:**
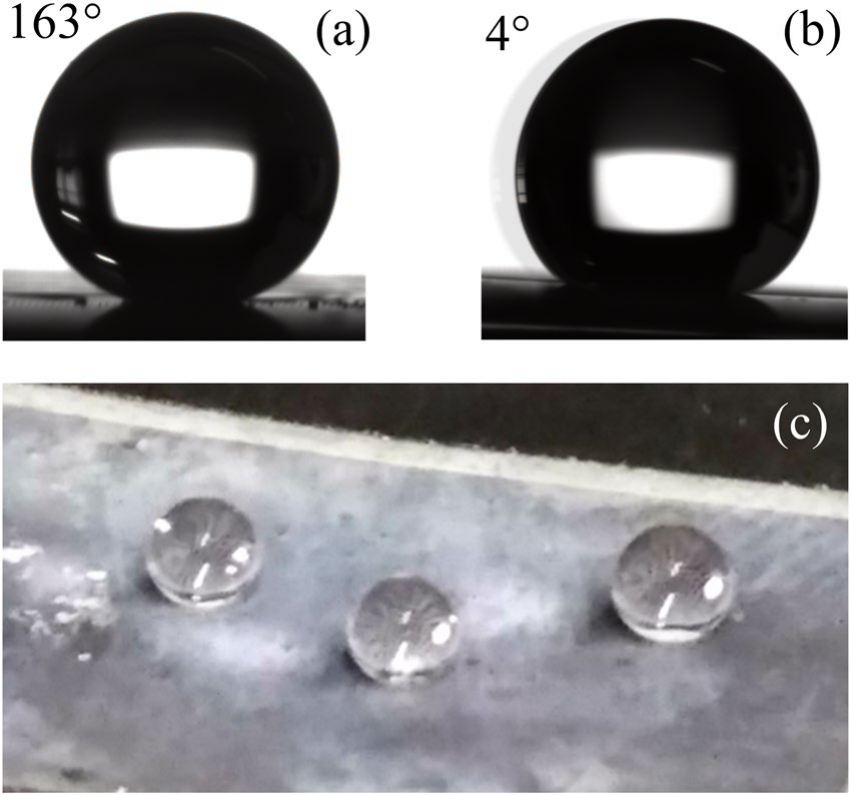
The contact angle (**a**), sliding angle (**b**) and optical image (**c**) of the processed surface.

The laser ablation process can generate microstructures on the surface of the sample. Growth of zinc oxide nanowires can further generate nanostructures on it. After the low surface energy material modification, the solid-liquid contact of the sample surface will be changed to Cassie state. Figure 6 shows the contact angle, sliding angle and optical image of the processed sample respectively. It can be seen that, the laser ablated surface with microstructures and ZnO nanowires shows low adhesion superhydrophobic properties. The water contact angle is 163° and the sliding angle is less than 4°. It can be expected that the processed surface with ZnO nanowires can be used for drag reduction.

### Mechanical durability analysis

**Figure 7:**
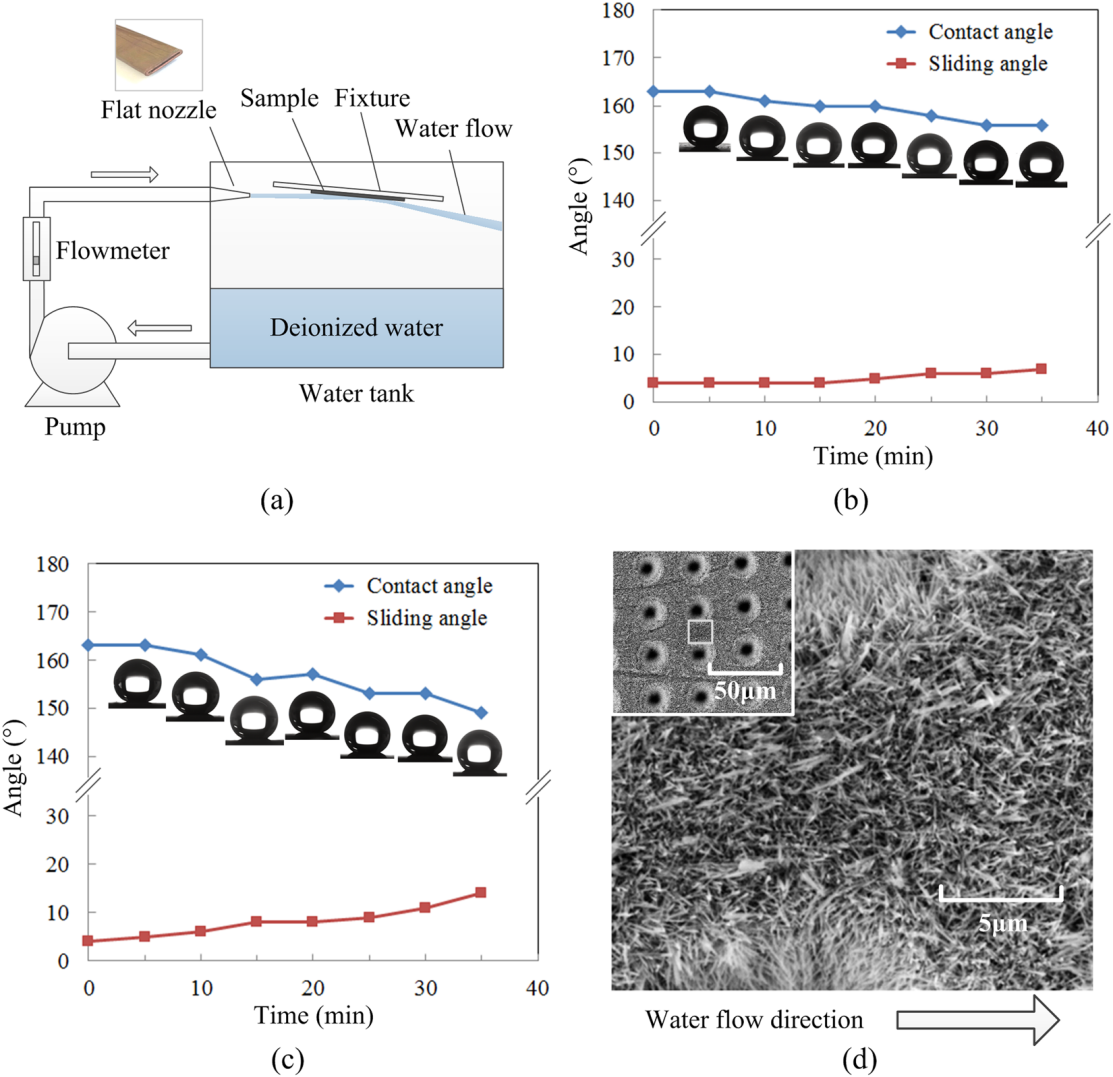
(**a**) Schematic illustration of water flow scouring test employed to evaluate the mechanical durability of the processed surface. (**b,c**) Evolutions of contact angle and sliding angle with water flow scouring time at 4m/s and 6m/s flow rate respectively. (**d**) SEM image showing the morphology of the surface after water flow scouring.

The mechanical durability test was carried out by water flow scouring method. The schematic of mechanical durability test is shown in Fig. 7a. The evolutions of contact angle and sliding angle with water flow scouring time at 4 m/s and 6 m/s flow rate are shown in Fig. 7b and c. The equivalent rotational speeds are 38000 rpm and 57000 rpm respectively.

It can be seen from Fig. 7b, during the experiment, the contact angle of the sample surface decreases gradually and the roll angle increases simultaneously. The changes in the experiment were reversible. When the sample was dried again after the experiment, the contact angle and sliding angle can be restored to 161° and about 5° respectively. Thus, when the water flow rate was 4 m/s, the hydrophobic surface was not destroyed during the experiment. This phenomenon can be explained by the escape of air bubbles from the micron-nano structures. This increases the solid-liquid contact area.

When the water flow rate increased to 6 m/s, the contact angle of the sample dropped sharply during the experiment and the sliding angle has more than tripled, as shown in Fig. 7c. After drying, the contact angle and sliding angle of the sample can only be restored to 153° and 9° respectively. This means that the hydrophobic surface has been damaged permanently during this test. In order to understand the changes in the surface of the sample, the SEM image showing the morphology of the surface (Fig. 7d) was observed again. It can be seen from the figure, after the impact of high-speed water flow, the zinc oxide nanowires on the sample surface tilted to the direction of flow and accompanied by shedding. The nanowires in the laser ablated microwells were almost unaffected.

### Mechanism of drag reduction

As shown in Fig. 1a, the water film in the spherical bearing is thin. By calculating the Reynolds number, it can be deduced that the fluid is in laminar flow state at all possible rotational speeds. The viscosity resistance torque of the liquid is the main resistance torque in the spherical bearing. Its value can be obtained by calculating the integral of the tangential viscous torque on the working surface of the spherical bearing, as is shown in Eq. (1).

1 $${T}_{{\rm{resist}}}={\int }_{0}^{2\pi }{\int }_{0}^{r}\eta \frac{{v}_{{\rm{d}}}}{h}{r}^{2}{\rm{d}}r{\rm{d}}\theta $$

In which, *T*_resist_ is the resistance torque of the spherical bearing, *r* is the maximum radius of the working surface, *η* is the viscosity of the fluid, *h* is the thickness of the water film, and *v*_d_ is the velocity difference between the upper and lower surfaces of the water film.

At low rotational speeds, the contact surface between the rotor ball and the water is in Cassie state. The low adhesion hydrophobic surface contains a large number of air bubbles inside. The actual solid-liquid contact area is greatly reduced, and becomes non-continuous. The solid-liquid interface exhibits a slip characteristic, reducing the flow rate difference of the upper and lower surface of the water film. When the rotor is rotating, the water flow will take the air bubbles away from the solid-liquid contact surface. Parts of the surface change to high adhesion Wenzel state. The drag reduction effect of the processed surface will decrease gradually during operation.

### Spherical bearing performance analysis

**Figure 8:**
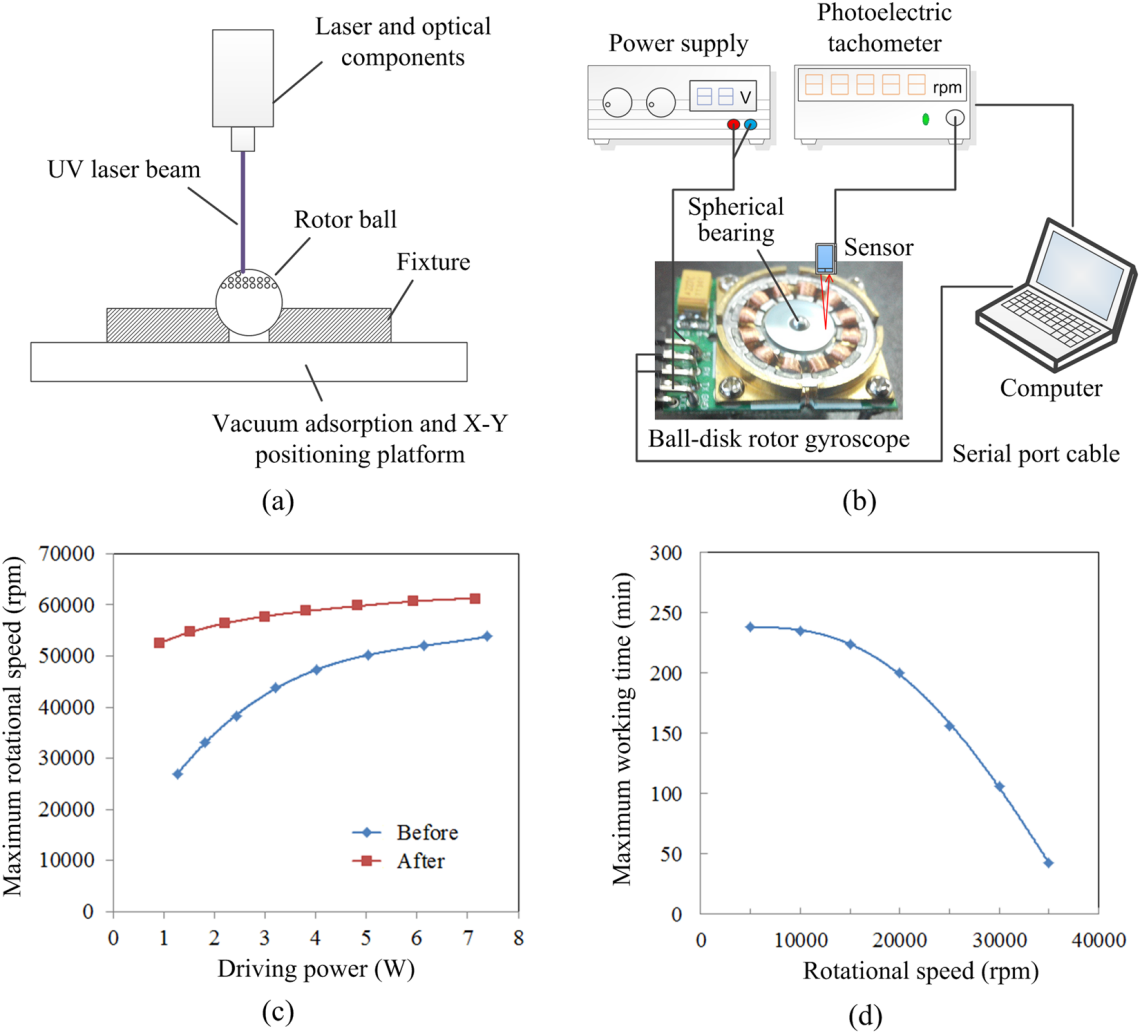
(**a**) Schematic of the laser ablation process on the rotor ball. (**b**) The block diagram of drag reduction test system. (**c**) Evolutions of maximum rotational speed with driving power. (**d**) Evolutions of maximum working time with rotational speed.

Laser ablation and ZnO nanowires growth method were applied to the ball in the spherical bearing to verify its drag reduction effect. Since the working surface of the spherical bearing is only the lower spherical crown about 2 mm in diameter, there is no need for uniform laser ablation of the entire sphere. It is impossible to achieve uniform laser ablation on the sphere with an X-Y positioning platform, the vertical laser ablation method was used instead on the spherical surface, as is shown in Fig. 8a. The block diagram of drag reduction test system is shown in Fig. 8b. In this system, we replaced the rotor ball with the treated one. A photoelectric tachometer was used to measure the rotational speed of the rotor. This experimental section contains two parts: the spinning-up test and the steady-state working time test.

The spinning-up tests were done under different driving voltages range from 5 V to 12 V, the corresponding driving power and the maximum rotational speeds were recorded. When the rotor is rotating at a constant speed, the driving torque balances with the resistance torque, and the drag reduction effect of the bearing can be deduced. Under each voltage, the target rotational speed is stepped up by 10 rpm every 5 ms until the rotor stalled. The test procedure was repeated 5 times to reduce random errors. The computer controlled the testing procedure through serial port and recorded the experimental data. The experimental results are shown in Fig. 8c. It can be seen from the figure, the maximum rotating speeds of the rotor at low driving power were significantly improved. When the driving power is 1.5 W, the maximum speed increases from 29950 rpm to 54740 rpm, the increasing rate is 82.77%. The equivalent drag reduction rate is 45.29%. But with the increase of the driving power, the increasing rate of the rotational speed decreases gradually.

The steady-state working time test is performed to evaluate the durability of the spherical bearing. In the mechanical durability test, it can be seen that the spherical bearing is not suitable for high speed operation such as 57000 rpm, and working speeds below 38000 rpm is reasonable. Thus, the steady-state working times at the rotational speed ranges from 5000 rpm to 35000 rpm were measured in the experiments. All these experiments were carried out at the same driving voltage of 5 V, and the experimental results are shown in Fig. 8d. It can be seen that the operating time of the device is more than 3.5 hours and changes gently when the speed is lower than 20000 rpm. When the rotational speed continues to increase, the device’s stable working time drops sharply. When the liquid flow rate is larger, it is easier to take away the air bubbles from the structure, causing the variation of the drag reduction effect.

## Conclusions

In this work, laser ablation and zinc oxide nanowires growth technologies were introduced into the spherical bearing for drag reduction applications. We have uniformly grown a layer of zinc oxide nanograss on a laser ablated microwells array. After the fluorination treatment, the sample surface with ZnO nanowires showed low adhesion superhydrophobic properties. The contact angle and the sliding angle of the surface are 163° and 4° respectively. The same treatment is applied to the spherical bearing of rotor gyroscope, and a good drag reduction effect can be achieved. The maximum rotating speed increasing rate is 82.77% at 1.5 W driving power. The equivalent drag reduction rate is 45.29% compared with the untreated spherical bearing. The experimental results show that the spherical bearing has good short term reliability. The rotational speed of ball-disk rotor gyroscope under 5 V driving voltage can be increased to 35000 rpm, and the normal operation time can be longer than 40 minutes. For the ball-disk rotor gyroscope is designed for short-term or one-time use, the stable working time is long enough to meet the requirements of the device. The superhydrophobic drag-reduction spherical bearing can increase the operating rotational speed of the ball-disk rotor gyroscope and improve the performance of the device.

## Experimental section

### Materials and preparation

Stainless steel foils (type 304), whose composition include C of <0.08 wt.%, Si of <1.0 wt.%, Mn of <2.0 wt.%, Cr of 18.0–20.0 wt.%, Ni of 8.0–10.0 wt.%, S of <0.030 wt.%, P of <0.045 wt.% and the remaining element Fe. Acetone (analytically pure), absolute ethylalcohol (analytically pure), deionized water, zinc acetate (analytically pure), zinc nitrate (analytically pure), hexamethylenetetramine (analytically pure), pure oxygen, 1-3-propanediamine (analytically pure), polyetherimide (30% solution in water), ethanol solution of fluorinated silane (0.5 wt.% 1 H,1 H,2 H,2H-perfluorooctadecyltrichlorosilane).

The stainless steel foils were cut into 2.5 × 2.5 cm^2^, ultrasonically cleaned in acetone, ethanol and deionized water successively to remove grease, and dried in a drying oven. Then microwell array of 30 μm pitch was etched on the surface of stainless steel foils using precision ultraviolet laser etching machine (Delphi FPS-V2). The scanning method was galvanometer scanning, the diameter of the laser spot was 10 μm and the power of the laser beam was 7 watts. The pulse width and frequency were 20 ns and 100 kHz respectively. The samples after laser ablation were ultrasonically cleaned in ethanol for 5 minutes, washed with deionized water and dried.

### Growth of ZnO nanowires

The laser ablated samples was immersed in 5 mM zinc acetate alcohol solution for 5 seconds and dried in a drying oven at 120 °C. This process was repeated three times. Then they were heated on the electric heating plate at 370 °C for 20 minutes. After this process, the sample surfaces were covered with a thin layer of zinc oxide, namely the seed layer for zinc oxide nanowires growth.

The reaction solution for ZnO nanowires growth was prepared in advance. The composition was 50 mM zinc nitrate, 25 mM Hexamethylenetetramine and 0.75 g PEI solution in 30 mL reaction solution. An appropriate amount of ammonia was dropped into the solution to adjust the pH of the mixed solution to the value of 8. Firstly, the reaction solution was preheated in the water bath to the reaction temperature of 88 °C. Then the stainless steel foils with zinc oxide seed layer were put into the reaction solution. The opening of the reaction vessel was then covered with plastic sheeting. The total reaction time was 8 hours. As the chemical reaction will generate ZnO both on the seed layer and in the bulk solution, consuming a large amount of the reactants, to keep the reasonable reaction rate, when the reaction time reached halfway, namely 4 hours, the reaction solution was changed once. When the reaction was completed, the samples were picked out from the reaction solution, rinsed with deionized water and then dried in a drying oven at 60 °C for half an hour.

Finally, the samples before and after ZnO nanowire growth were immersed in the ethanol solution of 0.5 wt.% 1 H,1 H,2 H,2H-perfluorooctadecyltrichlorosilane for 30 min respectively, and then dried in a drying oven. The similar processing methods were used on the stainless steel balls, and drag reduction experiments were done with them.

### Characterizations

The optical images of laser ablated surfaces were obtained by a 3D digital video microscope (Leica DVM2000). The surface morphologies were observed using a field-emission scanning electron microscope (TESCAN VEGA3), and the corresponding element distributions were determined by an energy-dispersive X-ray spectroscopy system (Oxford Instruments X-Max 50). The contact angles and sliding angles were measured by an optical contact angle meter (POWEREACH JC2000D2A). The mechanical durability and drag reduction performance were evaluated by a self-designed ball-disk rotor gyroscope test system.

### Mechanical durability test

To simulate the working state of the hydrophobic surface in the spherical bearing, the mechanical durability test was carried out by two sets of water flow scouring experiments. The flow rates were 4 m/s and 6 m/s respectively, corresponding to the case where the rotational speeds are 38000 rpm and 57000 rpm. The contact angle and sliding angle of the sample were measured every 5 minutes during each experiment.

## Electronic supplementary material

The effect of processing parameters on the quality of ZnO nanowires
