# Supplementary material for: Superhydrophobic Drag-Reduction Spherical Bearing Fabricated by Laser Ablation and PEI Regulated ZnO Nanowire Growth
Source: Sci Rep. 2017 Jul 20;7:6061. doi: 10.1038/s41598-017-05546-z (PMC5519682; doi:10.1038/s41598-017-05546-z)
Supplement: Supplementary file 1 — The effect of processing parameters on the quality of ZnO nanowires [file 41598_2017_5546_MOESM1_ESM.doc]

**Superhydrophobic Drag-Reduction Spherical Bearing Fabricated by Laser Ablation and PEI Regulated ZnO Nanowire Growth**

**Rui Weng^1^, Haifeng Zhang^1,2^*, Yanjing Tuo^1^, Yang Wang^1^, and Xiaowei Liu^1,2^**

^1^MEMS Center, Harbin Institute of Technology, Harbin, 150001, PR China

^2^State Key Laboratory of Urban Water Resource & Environment (Harbin Institute of Technology), Harbin 150001, China

*zhanghf@hit.edu.cn

**Contents:**

**1. Supplementary Discussion:**

**The effect of processing parameters on the quality of ZnO nanowires.**

**Supplementary Discussion:**

**The effect of processing parameters on the quality of ZnO nanowires.**

**Effect of Zinc Concentration on Surface Morphology**

The other conditions were kept constant, and experiments with zinc nitrate concentration of 10mM, 20mM and 50mM were done respectively. Then the surface morphologies of ZnO nanowires were observed, as shown in Supplementary Fig 1.


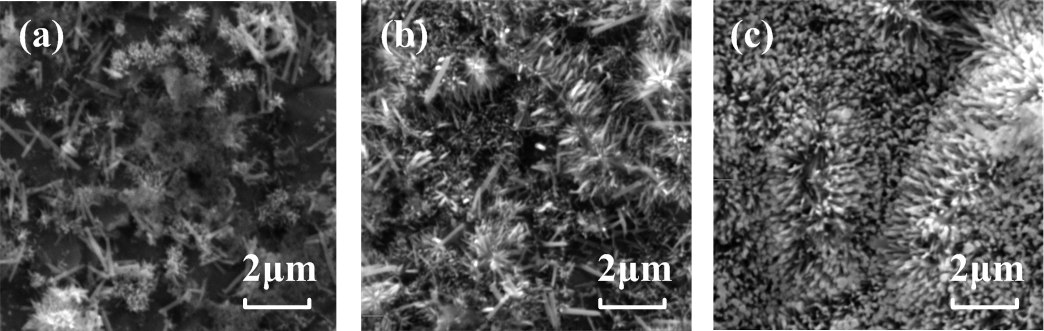


Supplementary Figure 1. The surface morphologies of ZnO nanowires when the concentration of zinc nitrate was 10mM (a), 20mM (b) and 50mM (c).

As can be seen from the figure, when the concentration of zinc nitrate increases, the resulting nanowires become dense, so it is reasonable to select a higher concentration of zinc nitrate solution.

**Effect of the amount of PEI solution on Surface Morphology**

The other conditions were kept constant, and experiments with 0.35g, 0.75g and 1.50g PEI solution were done respectively. Then the surface morphologies of ZnO nanowires were observed, as shown in Supplementary Fig 2.


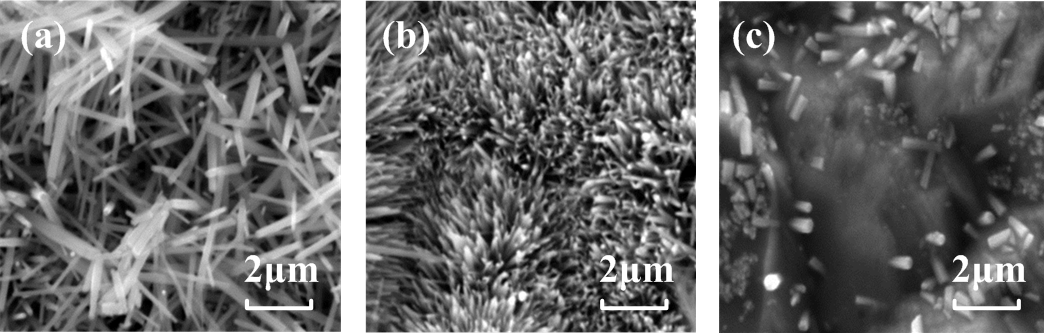


Supplementary Figure 2. The surface morphologies of ZnO nanowires when the amount of PEI solution was 0.35g (a), 0.75g (b) and 1.50g (c).

It can be seen from the figure, when the PEI concentration was low, the generated nanowires were long but sparse. When the amount of PEI was moderate, the length of the nanowires was uniform and neat, and had good hydrophobicity after fluorination. When the PEI solution was excessive, the nanowires were shorter and thicker, and it showed a cluster phenomenon. After fluoride, the surface hydrophobicity was low.

**Effect of the pH value of solution on Surface Morphology**

The other conditions were kept constant, and experiments with the pH value of 8, 10 and 11 were done respectively. Then the surface morphologies of ZnO nanowires were observed, as shown in Supplementary Fig 3.


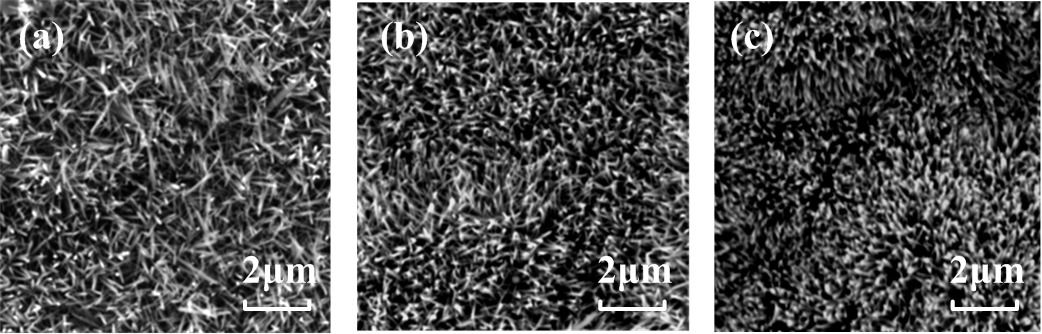


Supplementary Figure 3. The surface morphologies of ZnO nanowires when the pH value was
8 (a), 10 (b) and 11 (c).

As can be seen from the figure, with the increase of pH value, the density of ZnO nanowires gradually increased. But the diameter of the nanowires also increased. After the fluorination treatment, the best hydrophobic effect occurs at pH 8. So we choose 8 as the optimum pH value.
